# Macrophage inhibitory cytokine 1, syncollin and thrombospondin-2 in pancreatic ductal adenocarcinoma and chronic pancreatitis differentiation

**DOI:** 10.3389/fonc.2026.1866001

**Published:** 2026-06-26

**Authors:** Michalina Wieczorek, Lukasz Wieczorek, Anna Borkowska, Ewa Malecka-Wojciesko

**Affiliations:** 1 Department of Digestive Tract Diseases, Medical University of Lodz, Lodz, Poland; 2 Institute of Information Technology, Lodz University of Technology, Lodz, Poland

**Keywords:** chronic pancreatitis, macrophage inhibitory cytokine 1, pancreatic ductal adenocarcinoma, serum biomarkers, syncollin, thrombospondin-2

## Abstract

**Introduction:**

Pancreatic ductal adenocarcinoma (PDAC) is a highly lethal malignancy with a rising global incidence. Differentiating PDAC from non-malignant pancreatic conditions, particularly chronic pancreatitis (CP), remains challenging due to overlapping clinical and radiological features, highlighting the need for new biomarkers. The best-validated serum biomarker, carbohydrate antigen 19-9 (CA19-9), has limited clinical utility due to its suboptimal sensitivity and specificity. This study aimed to evaluate the diagnostic performance of serum macrophage inhibitory cytokine 1 (GDF15), syncollin (SYCN), and thrombospondin-2 (TSP-2), both alone and in multi-marker panels, for differentiating PDAC from CP and healthy controls (HCs). The selection of these markers was based on prior evidence linking GDF15 to PDAC diagnosis and prognosis, SYCN to pancreatic tissue damage, and TSP-2 to tumor microenvironment remodeling.

**Methods:**

This study included 188 individuals: 78 diagnosed with PDAC, 79 with CP and 31 HCs. PDAC and CP were diagnosed based on clinical, imaging, histopathological, and laboratory findings, and classified according to the 8th TNM classification and the updated Cambridge system, respectively. Serum GDF15, SYCN, and TSP-2 levels were quantified using ELISA. Statistical analyses included group comparisons, correlation testing, and receiver operating characteristic (ROC) curve analysis with assessment of the area under the curve (AUC).

**Results:**

GDF15 and SYCN were significantly elevated in PDAC compared with both CP and HCs, whereas TSP-2 did not differ significantly between those groups. For PDAC vs HCs, GDF15 provided the strongest overall discrimination (AUC = 0.86; sensitivity 97%, specificity 71%), while SYCN showed a lower AUC (0.77) but very high specificity (94%). The combined GDF15 + SYCN panel increased AUC to 0.89. For PDAC vs CP, GDF15 achieved moderate diagnostic performance (AUC = 0.73), SYCN performed less well (AUC = 0.65), and TSP-2 performed near chance (AUC = 0.52). Incorporating age and bilirubin in the model improved discrimination between PDAC and CP, yielding a maximum AUC of 0.88. Additionally, positive correlations were observed between SYCN and diabetes, as well as between GDF15 and hyperbilirubinemia, in patients with PDAC.

**Conclusions:**

These results suggest that GDF15 and SYCN are promising serum biomarkers for PDAC, whereas TSP-2 appears to have limited diagnostic utility.

## 1 Introduction

Pancreatic ductal adenocarcinoma (PDAC) is a malignancy characterized by low survival rates and progressively increasing global incidence. In Poland, approximately 3,800 new cases are diagnosed annually. The contribution of PDAC to the overall years of potential life lost due to cancer has steadily risen in recent years, currently surpassing 5% (1). The poor prognosis of PDAC is largely related to delayed diagnosis and the frequent detection of disease at an advanced or metastatic stage. Metastatic dissemination most commonly involves the liver, lungs, and peritoneum, but also, less frequently, the bones (2). This further illustrates the biological heterogeneity of PDAC and supports the need for integrated diagnostic strategies combining clinical assessment, imaging, and biomarker-based approaches. An important clinical challenge lies in the accurate differentiation of PDAC from non-malignant pancreatic conditions, including chronic pancreatitis (CP) and autoimmune pancreatitis. Despite advances in radiological methods, imaging alone remains inadequate for a definitive PDAC diagnosis, particularly in the context of mass-forming CP (3, 4). The clinical presentation of malignant and benign pancreatic diseases (e.g. jaundice, abdominal pain, weight loss, diarrhea, fatigue) and complications like diabetes or exocrine insufficiency may present with overlapping clinical manifestations. Therefore, there is a need for specific biomarkers that could support the diagnosis of PDAC and its differentiation from non-neoplastic pancreatic conditions (5–10). In this context, additional circulating biomarkers may be useful as complementary diagnostic tools. Accordingly, the aim of the present study was to evaluate the preliminary diagnostic potential of selected circulating biomarkers in patients with PDAC and to assess their ability to support differentiation from non-malignant pancreatic conditions and healthy controls (HCs).

Serum carbohydrate antigen 19-9 (CA19-9) has been the most validated and widely used PDAC biomarker, which demonstrates utility as a prognostic tool, facilitating the assessment of surgical treatment response or indicating on the unresectable, advanced disease (11, 12). Nevertheless, its low specificity and absence in Lewis antigen negative subjects limits its clinical use both in PDAC diagnosis as well as its differentiation from numerous benign conditions of the pancreas, liver or the biliary tract, where CA19–9 may be also elevated (11, 13). The routine evaluation of serum CA19–9 level lacks efficacy as a mass screening method in asymptomatic individuals, owing to its limited sensitivity in early-stage disease (13–15).

Macrophage inhibitory cytokine 1 (GDF15) is a member of transforming growth factor-β (TGF-β) super-family of proteins correlating with stress-induced pathways and macrophage activation (16). Multiple studies in human and animal models demonstrate that GDF15 serum concentration rises in the context of inflammation, tissue injury or cellular stress and have been observed in numerous conditions such as: cardiovascular diseases (including ischemia, myocardial infarction, stroke), diabetes or liver cirrhosis (17–20). Crucially, accumulating data suggest a regulatory role of GDF15 in tumorigenesis, which may be linked to its p53-responsive promoter region (21). Among 240 oncological patients, mean GDF15 levels were preoperatively assessed, revealing the highest concentrations in PDAC cases (1731.0 ± 1181.0 pg/mL) in comparison to 8 other cancers, i.a., non-small-cell lung carcinoma (1258.0 ± 587.3 pg/mL; p=0.029) and colorectal adenocarcinoma (1371.0 ± 818.7 pg/mL; p=0.069) (22).

The pancreas-enriched protein syncollin (SYCN) was first identified in 1997, and its localization to the luminal surface of pancreatic zymogen granule membranes was subsequently determined using SDS-PAGE, immunoblotting, and *in vitro* translation (23, 24). In experimental models of SYCN knockout mice, compensatory pancreatic hypertrophy, elevated digestive enzyme levels, and altered enzyme trafficking indicated an essential role for SYCN in exocrine pancreatic exocytosis, as well as in the efficient formation and maturation of zymogen granules (25, 26). Moreover, SYCN was detected in pancreatic juice samples from PDAC patients using a comprehensive proteomic approach based on liquid chromatography-tandem mass spectrometry (27). To the best of our knowledge, only two human studies have investigated SYCN in PDAC to date, reporting elevated serum levels of this protein (28, 29). Disruption of the exocrine pancreatic architecture and function in PDAC may promote the release of zymogen granule proteins, such as SYCN, into pancreatic juice and the circulation. This interpretation is supported by evidence from transgenic mice overexpressing TGF-α, in which acinar cells enriched in zymogen granules underwent acinar-to-ductal transdifferentiation and progressed toward malignancy (30).

Thrombospondin-2 (TSP-2) is a matricellular protein playing a crucial role in cell-matrix communication, endothelial cells migration process and the modulation of apoptosis–proliferation balance (31, 32). Tumor-associated vascularization was markedly elevated in TSP-2-deficient mice compared with wild-type controls, highlighting the inhibitory effect of this biomarker on angiogenesis (33). In another study, a pan-cancer analysis of 33 tumor types using transcriptomic data and computational methods showed that TSP-2 expression correlates positively with immunosuppressive M2 macrophages, negatively with tumor-suppressive subsets, and is associated with over 30 immune checkpoint genes in 20 malignancies, including PDAC (34).

The current exploratory study aims to provide a preliminary evaluation of the diagnostic value of GDF15, SYCN, and TSP-2 in PDAC and their utility in distinguishing PDAC from CP.

## 2 Methods

This study included 188 individuals: 78 diagnosed with PDAC, 79 with CP, and 31 HCs. All participants were hospitalized at the Department of Digestive Tract Diseases of the Medical University of Lodz. The HC group consisted of patients admitted for one-day hospitalization for diagnostic evaluation of gastrointestinal symptoms, in whom endoscopic assessment revealed no abnormalities. Based on the medical interview, HCs denied any history of conditions potentially affecting biomarker levels, including chronic inflammatory diseases, inflammatory bowel disease, liver disease, and malignancy. The final sample size was determined by the availability of eligible participants with complete clinical data and serum samples suitable for ELISA-based biomarker measurements during the study period. CA19–9 was not included in the comparative analyses because complete and methodologically consistent CA19–9 data were not available across the entire cohort. Therefore, the analysis was limited to selected biomarkers measured consistently by ELISA. The characteristics of the study groups, including gender distribution, mean age, and body mass index (BMI), are presented in **Table 1**. Additional clinical data regarding alcohol consumption patterns, tobacco use, and the presence of comorbidities were also collected. For the purpose of this study, elevated bilirubin was defined as a total serum bilirubin concentration > 1.5 mg/dL, whereas regular alcohol consumption was assessed based on patient-reported history obtained during the medical interview and classified as present or absent.

**Table 1:** Characteristics of the study groups.

Clinical variable	HCs	CP	PDAC
No. of patients, n	31	79	78
Male sex, (%)	9 (29.03%)	55 (69.62%)	31 (39.74%)
Mean age (years)	68.52 ± 14.86	57.15 ± 12.72	69.25 ± 10.39
Mean BMI (kg/m^2^)	NA	22.48 ± 4.46	23.56 ± 3.19
Ever smokers	NA	56 (70.89%)	24 (30.77%)
Diabetes	NA	27 (34.18%)	26 (33.33%)
Regular alcohol consumption	NA	26 (32.91%)	3 (3.85%)
Mean bilirubin level (mg/dL)	NA	0.99 ± 0.93	6.48 ± 7.16
Hyperbilirubinemia	NA	6 (7.59%)	47 (60.26%)

HCs, healthy controls; CP, chronic pancreatitis; PDAC, pancreatic ductal adenocarcinoma; BMI, body mass index; NA, not available; Hyperbilirubinemia– serum bilirubin level >1,5 mg/dl.

The final PDAC diagnosis was based on imaging techniques, including CT and MRI, and was confirmed by histopathological analysis of tissue obtained either via endoscopic ultrasound-guided fine-needle biopsy (EUS-FNB) or from postoperative surgical specimens. Tumor staging was performed according to the 8th AJCC/UICC TNM classification (35). Most patients with PDAC were at TNM stage IV (N = 53; 67.95%), while 10 patients (12.82%) were classified as stage III and 15 (19.23%) had early-stage disease, corresponding to TNM stages I–II (**Table 2**). Details regarding prior or ongoing chemotherapy, laboratory parameters, accompanying diabetes, impaired glucose tolerance, and other relevant clinical data were also collected. In patients with CP, diagnosis was based on a combination of clinical manifestations, patient history, and typical imaging findings, including pancreatic calcifications. Additional functional and metabolic data, including decreased elastase-1 levels and abnormal fasting glucose levels, were collected to characterize exocrine pancreatic insufficiency, impaired glucose tolerance, or overt diabetes. The severity of CP on imaging techniques was determined using the updated Cambridge classification system (36–38), with grade distribution presented in **Table 3**.

**Table 2:** Tumor stage according to 8th TNM classification in pancreatic ductal adenocarcinoma.

TNM classification	PDAC patients (n = 78)
*IA*	2 (2.56%)
*IB*	6 (7.69%)
*IIA*	1 (1.28%)
*IIB*	6 (7.69%)
*III*	10 (12.82%)
*IV*	53 (67.95%)

**Table 3:** Chronic pancreatitis advancement according to Cambridge classification.

Cambridge classification	CP patients (n = 79)
*0*	2 (2.53%)
*1*	10 (12.66%)
*2*	19 (24.05%)
*3*	10 (12.66%)
*4*	38 (48.10%)

Patients diagnosed with malignancies other than PDAC, those who had received oncological treatment for non-PDAC malignancies within the preceding 10 years, and patients with chronic inflammatory conditions, such as inflammatory bowel diseases or chronic liver disease, were excluded from the study. Prior or ongoing treatment for PDAC or CP did not preclude participation in the study. Information on treatment status at the time of enrollment was recorded in the study database based on medical history and available medical records.

Venous blood samples were collected into sterile serum collection tubes and processed by centrifugation at 3000 rpm within 30 minutes. After centrifugation, the separated serum was stored at −80 °C until biomarker analysis. All measurements were performed on the same day, under comparable laboratory conditions, by a single trained operator. Serum concentrations of GDF-15, SYCN, and TSP-2 were measured using commercially available human enzyme-linked immunosorbent assay (ELISA) kits from Biorbyt Ltd. (Cambridge, UK): Human GDF-15 ELISA Kit (cat. no. orb561751), Human SYCN/Syncollin ELISA Kit (cat. no. orb564551), and Human TSP-2/THBS2 ELISA Kit (cat. no. orb50136). All assays were performed according to the manufacturer’s instructions. The assays were based on a sandwich ELISA format using pre-coated 96-well plates, biotinylated detection antibodies, and horseradish peroxidase (HRP)-mediated colorimetric detection. Briefly, standards and serum samples were added to the wells and incubated according to the kit protocols. After the addition of detection antibodies and HRP-conjugated reagents, the plates were washed, TMB substrate was added, and the enzymatic reaction was stopped. Absorbance was measured at 450 nm, and biomarker concentrations were calculated from kit-specific standard curves. According to the manufacturer’s documentation, the analytical ranges were 625–40,000 pg/mL for TSP-2, 23.438–1,500 pg/mL for GDF-15 and 0.156–10 ng/mL for SYCN. The reported analytical sensitivities were <10 pg/mL, 14.063 pg/mL and 0.094 ng/mL, respectively. Manufacturer-reported intra-assay and inter-assay coefficients of variation were below 10% for all three kits. Biomarker distributions were inspected for extreme values, and no observations were removed unless technical or measurement error was identified.

The study protocol was approved by the Bioethics Committee of the Medical University of Lodz (approval no. RNN43/23/KE). Statistical analyses were performed using Python, employing standard scientific libraries including pandas, numpy, scipy, matplotlib, and scikit-learn. Normality of distributions was evaluated using the Shapiro–Wilk test. Then, depending on data characteristics, group comparisons were conducted using either ANOVA or the Kruskal–Wallis test for continuous variables, and the chi-squared test for categorical variables. Pairwise analyses were carried out using the Student’s t-test or the Mann–Whitney U test, based on distribution normality and variance homogeneity. A p-value below 0.05 was considered statistically significant. Data are presented as medians with interquartile ranges (IQR; Q1–Q3). To account for multiple testing, predefined pairwise biomarker comparisons were corrected using the Holm method. Both unadjusted and Holm-adjusted p-values were reported. Correlation analysis was conducted using Spearman’s rank correlation coefficient due to the non-normal distribution of the analyzed variables. For associations between continuous variables and dichotomous clinical variables, point-biserial correlation was applied. Finally, receiver operating characteristic (ROC) curves and area under the curve (AUC) values were calculated using scikit-learn to assess the diagnostic performance of the biomarkers, including sensitivity (Se) and specificity (Sp). Optimal diagnostic thresholds were determined using Youden’s index, calculated as sensitivity + specificity − 1. For individual biomarkers, cut-offs were reported as the corresponding biomarker concentrations. For multivariable panels, biomarkers and clinical covariates were combined using logistic regression models, and model-derived predicted probabilities were used for ROC curve analysis. Therefore, panel cut-offs represent Youden-optimal probability thresholds for the whole model, not thresholds for individual biomarkers or clinical variables. Internal validation of the best-performing multivariable logistic regression models was performed using bootstrap resampling. Optimism-corrected AUC values and bootstrap-derived 95% confidence intervals were calculated to assess model stability.

## 3 Results

**Figure 1:**
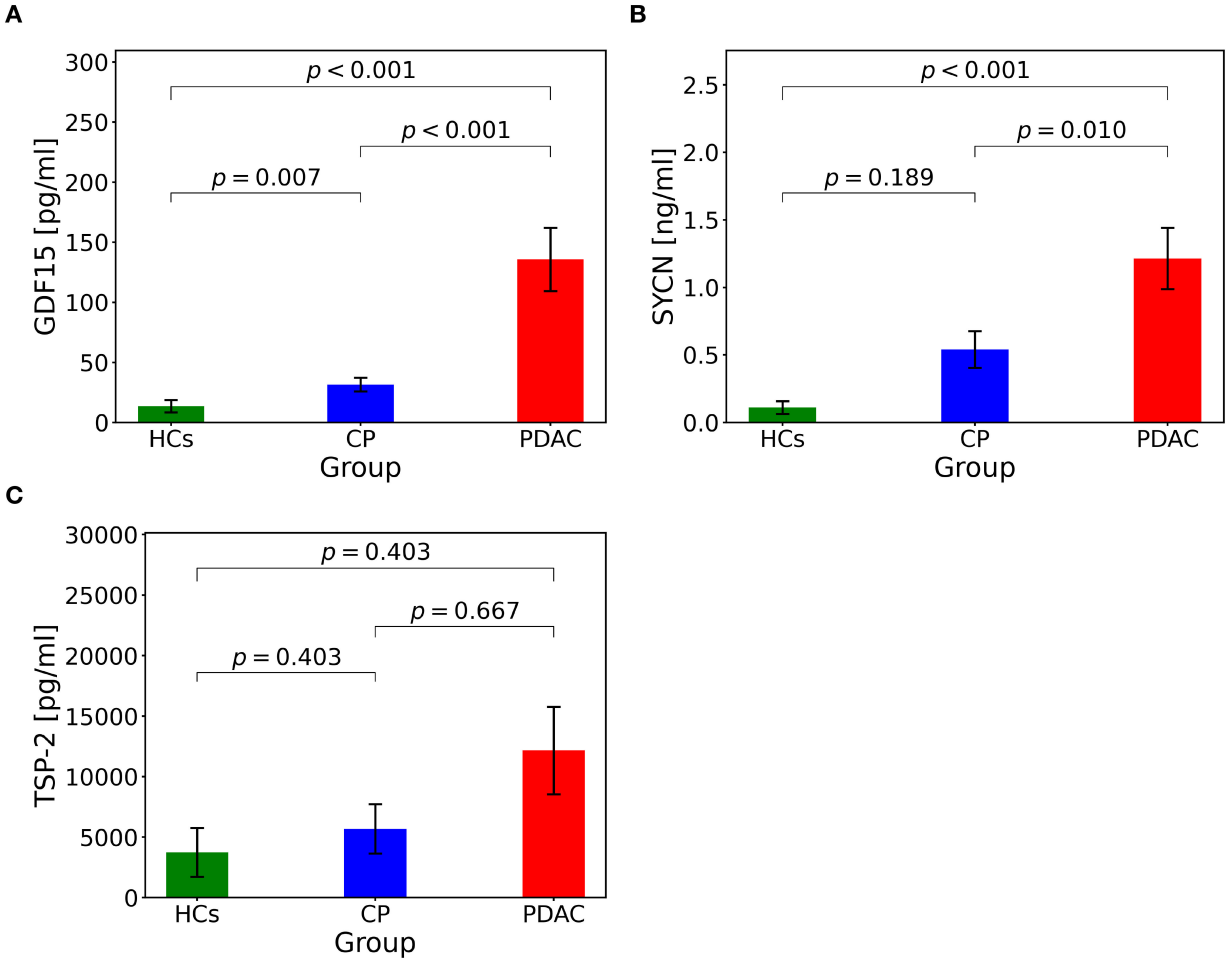
Serum concentrations of GDF15 **(A)**, SYCN **(B)** and TSP-2 **(C)** in PDAC, CP, and HCs.

Median GDF15 levels were higher in patients with PDAC (Me = 36.2 pg/mL, IQR: 12.73–151.86) compared with both HCs (Me = 0.63 pg/mL, IQR: 0.39–12.2) and patients with CP (Me = 11.34 pg/mL, IQR: 6.24–25.58). After Holm correction for multiple comparisons, GDF15 remained significantly different in all predefined pairwise group comparisons, including PDAC versus HCs and PDAC versus CP (adjusted p < 0.001 for both comparisons). Similarly, SYCN levels were markedly elevated in the PDAC group (Me = 0.38 ng/mL, IQR: 0.04–1.41) compared with HCs (Me = 0.03 ng/mL, IQR: 0.03–0.06) and patients with CP (Me = 0.06 ng/mL, IQR: 0.03–0.43). After Holm correction, SYCN remained significantly different in comparisons involving PDAC, including PDAC versus HCs (adjusted p < 0.001) and PDAC versus CP (adjusted p < 0.01), whereas the HCs versus CP comparison was not significant (adjusted p = 0.19). In contrast, serum TSP-2 levels were comparable across the examined groups: PDAC (Me = 70.50 pg/mL, IQR: 45.15–369.65), CP (Me = 67.60 pg/mL, IQR: 44.15–282.76), and HCs (Me = 58.70 pg/mL, IQR: 44.2–65.35). None of the predefined pairwise comparisons for TSP-2 remained statistically significant after Holm correction (**Figure 1**; **Table 4**).

**Table 4:** Mean serum, IQR, median and range of GDF15, syncollin and TSP-2 in patients with PDAC, CP and HCs.

Study group	Biomarker	Mean ± STD	IQR	Min	25%	Median	75%	Max
HCs	GDF15 [pg/mL]	13.62 ± 28.31	11.81	0.17	0.39	0.63^(a)(e)^	12.2	137.91
	SYCN [ng/mL]	0.11 ± 0.27	0.03	0.01	0.03	0.03^(b)(f)^	0.06	1.13
	TSP-2 [pg/mL]	3731.33 ± 11272.69	21.15	21.60	44.20	58.7	65.35	53977.0
CP	GDF15 [pg/mL]	31.59 ± 50.647	19.34	0.19	6.24	11.34^(c)(e)^	25.58	251.4
	SYCN [ng/mL]	0.54 ± 1.21	0.4	0.002	0.03	0.06^(d)(f)^	0.43	7.26
	TSP-2 [pg/mL]	5674.11 ± 18150.76	238.63	19.5	44.15	67.6	282.78	92948.0
PDAC	GDF15 [pg/mL]	135.74 ± 232.09	139.13	1.67	12.76	36.2^(a)(c)^	151.86	1385.3
	SYCN [ng/mL]	1.21 ± 2.01	1.37	0.01	0.04	0.38^(b)(d)^	1.41	12.18
	TSP-2 [pg/mL]	12154.14 ± 31959.6	334.5	16.4	45.15	70.5	379.65	157090.0

Significant differences between groups: (a) Raw p and Holm-adjusted p < 0.001 for GDF15: HCs vs PDAC, (b) Raw p and Holm-adjusted p < 0.001 for SYCN: HCs vs PDAC, (c) Raw p and Holm-adjusted p < 0.001 for GDF15: CP vs PDAC, (d) Raw p = 0.002 and Holm-adjusted p = 0.009 for SYCN: CP vs PDAC, (e) Raw p < 0.001 and Holm-adjusted p = 0.007 for GDF15: HCs vs CP, (f) Raw p = 0.038, but Holm-adjusted p = 0.19 for SYCN: HCs vs CP;

HCs, healthy controls; CP, chronic pancreatitis; PDAC, pancreatic ductal adenocarcinoma; GDF15, growth differentiation factor 15; SYCN, syncollin; TSP-2, thrombospondin-2; STD, standard deviation, IQR, interquartile range, Raw p, unadjusted p-value.

Furthermore, biomarker concentrations were analyzed in relation to selected clinical and demographic parameters. An exploratory stage-stratified analysis was performed to compare biomarker levels in HCs and CP patients with those observed in early-stage and advanced-stage PDAC. To allow stage-based comparisons, stages I–II were combined as early-stage PDAC and stages III–IV as advanced-stage PDAC. When HCs were used as the reference group, median GDF15 levels were 40.52 pg/mL in early-stage PDAC and 34.90 pg/mL in advanced-stage PDAC. After Holm correction, GDF15 concentrations were significantly higher in both early-stage and advanced-stage PDAC compared with HCs (adjusted p < 0.001 for both comparisons). Median SYCN levels were 0.30 ng/mL in early-stage PDAC and 0.40 ng/mL in advanced-stage PDAC. SYCN concentrations were significantly higher in advanced-stage PDAC compared with HCs (adjusted p < 0.001), while the difference between HCs and early-stage PDAC was not significant after correction (adjusted p = 0.10). TSP-2 concentrations were comparable across these groups, with median levels of 75.20 pg/mL in early-stage PDAC and 65.80 pg/mL in advanced-stage PDAC; none of the pairwise comparisons remained statistically significant after Holm correction. When CP was used as the reference group, GDF15 concentrations were significantly higher in both early-stage and advanced-stage PDAC compared with CP after Holm correction (adjusted p = 0.01 and adjusted p < 0.001, respectively). SYCN concentrations were significantly higher in advanced-stage PDAC compared with CP (adjusted p < 0.01), while the difference between CP and early-stage PDAC was not significant after correction (adjusted p = 0.40). TSP-2 concentrations were also comparable across CP, early-stage PDAC, and advanced-stage PDAC, with no significant pairwise comparisons after Holm correction. No significant differences in GDF15, SYCN, and TSP-2 concentrations were observed between early-stage and advanced-stage PDAC.

Among PDAC patients with hyperbilirubinemia, defined as bilirubin > 1.5 mg/dL, median GDF15 concentration was significantly higher than in patients without hyperbilirubinemia (92.15 pg/mL, IQR: 23.57–175.60, n = 47 vs. 15.33 pg/mL, IQR: 10.88–39.02, n = 31; p < 0.01). No statistically significant differences were observed for SYCN or TSP-2 between PDAC patients with and without hyperbilirubinemia. Median SYCN concentrations were 0.45 ng/mL (IQR: 0.05–1.57) in patients with hyperbilirubinemia and 0.27 ng/mL (IQR: 0.04–0.82) in those without hyperbilirubinemia (p = 0.41). Corresponding TSP-2 concentrations were 63.20 pg/mL (IQR: 37.80–369.00) and 99.50 pg/mL (IQR: 54.15–1967.00), respectively (p = 0.22).

**Figure 2:**
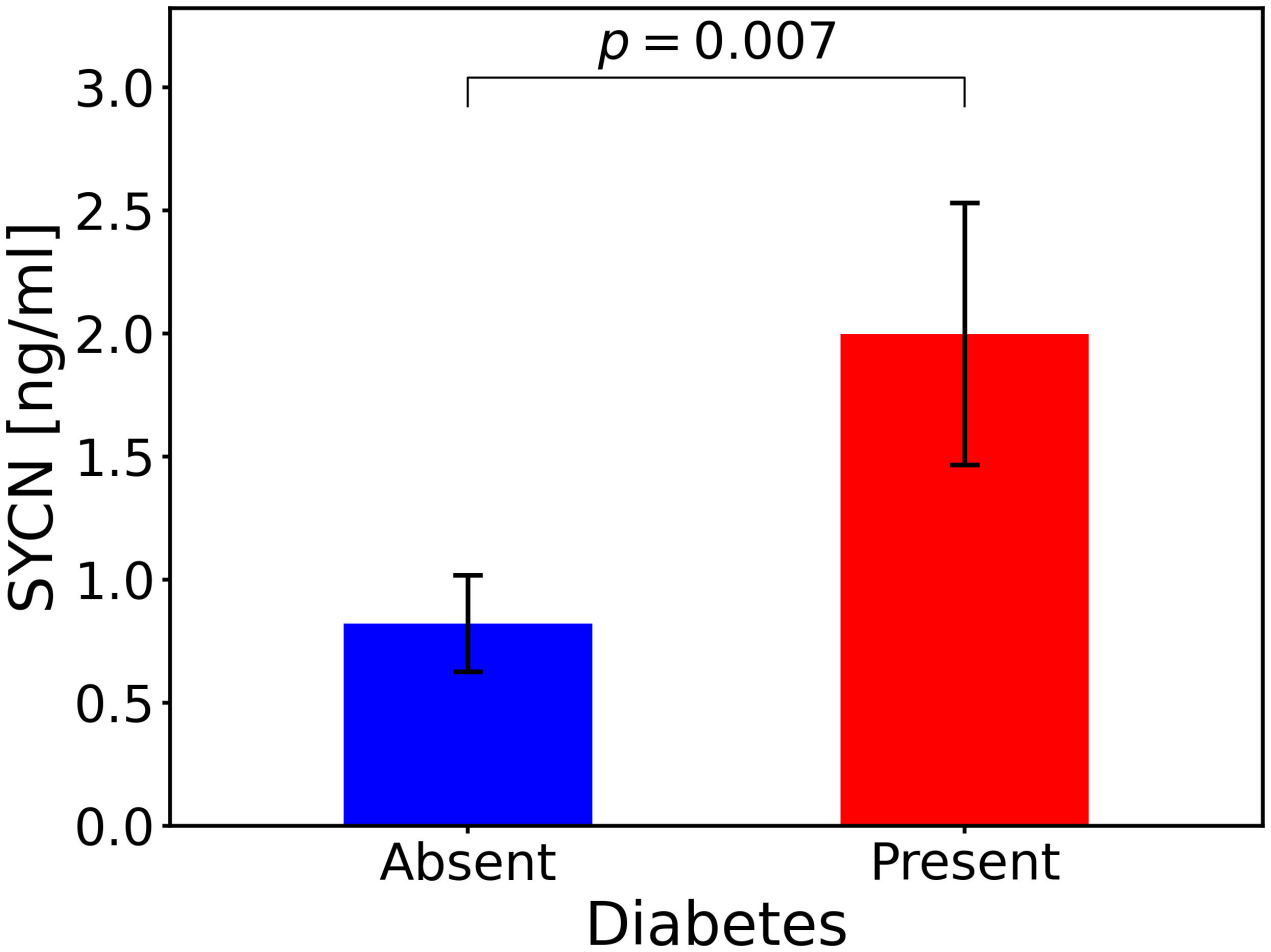
Serum SYCN concentrations in PDAC patients with and without diabetes.

In addition, median SYCN concentrations were significantly higher in PDAC patients with concomitant diabetes (n = 26; Me = 1.31 ng/mL, IQR: 0.33–2.05) compared with those without glucose tolerance impairment (n = 52; Me = 0.23 ng/mL, IQR: 0.04–0.77; p < 0.01; **Figure 2**). No statistically significant differences in GDF15 or TSP-2 concentrations were observed between PDAC patients with and without concomitant diabetes. Median GDF15 concentration was 54.72 pg/mL (IQR: 16.13–172.78) in patients with diabetes and 33.3 pg/mL (IQR: 11.56–132.47) in those without glucose tolerance impairment (p = 0.27). Corresponding TSP-2 concentrations were 58.05 pg/mL (IQR: 44.90–4333) and 78.90 pg/mL (IQR: 49.30–333.60) in these two subgroups, respectively (p = 0.77).

In the CP group, serum TSP-2 levels were significantly higher in patients reporting regular alcohol consumption than in those without such history (Me = 223.20 pg/mL, IQR: 51.12–3797 vs. Me = 64.3 pg/mL, IQR: 42.7–122.2; p = 0.03). No analogous association was observed for GDF15 or SYCN. Median GDF15 concentrations were 11.29 pg/mL (IQR: 6.97–62.22) in patients reporting regular alcohol consumption and 11.34 pg/mL (IQR: 6.3–18.32) in the remaining CP patients (p = 0.60). For SYCN, median concentrations were 0.06 ng/mL (IQR: 0.03–0.28) and 0.06 ng/mL (IQR: 0.03–0.44), respectively (p = 0.90).

No statistically significant differences in GDF15, SYCN, or TSP-2 concentrations were found according to smoking status, BMI category, or chemotherapy status.

**Figure 3:**
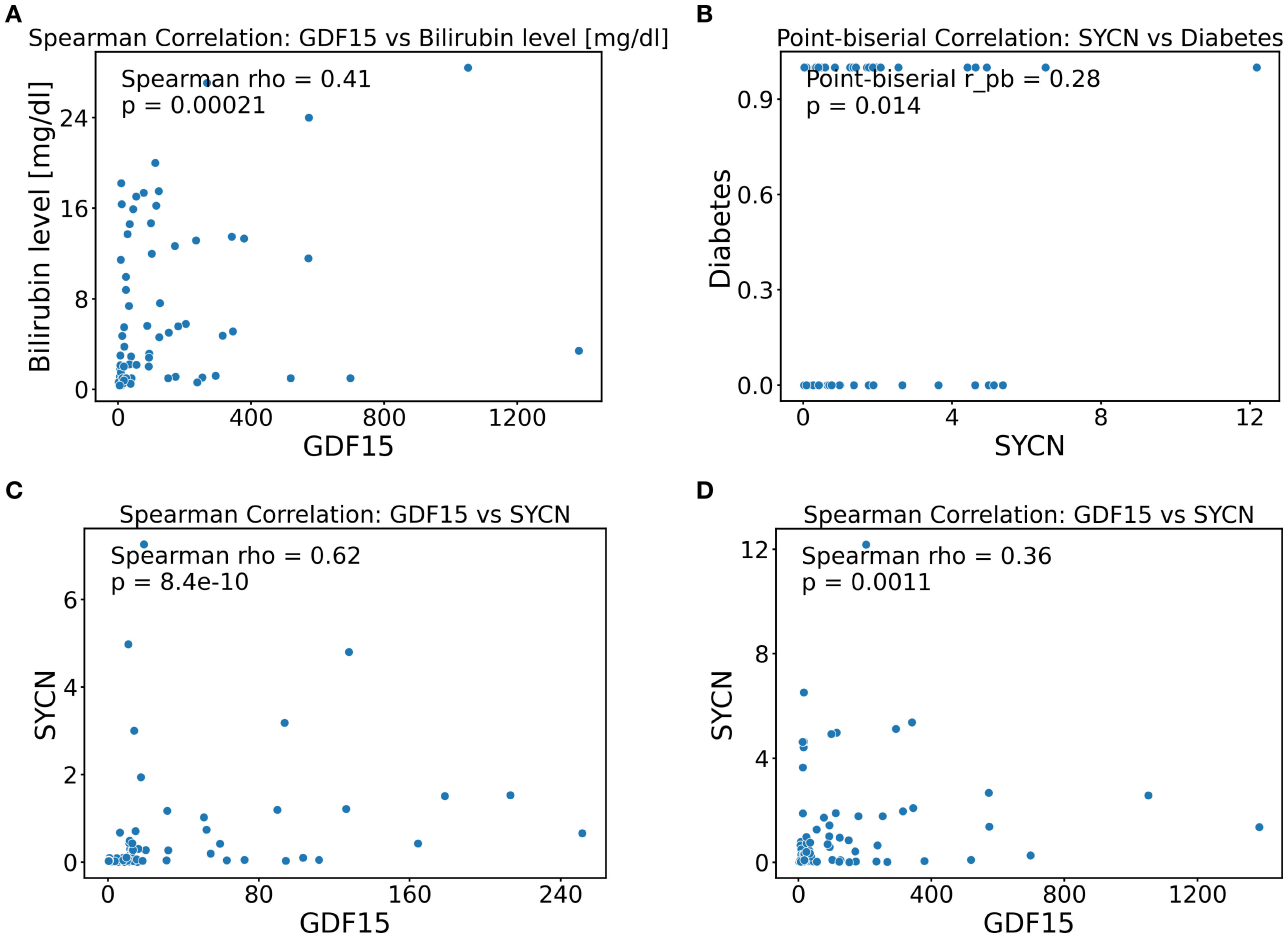
**(A)** Scatter plot of GDF15 versus serum bilirubin in PDAC patients. **(B)** Scatter plot of SYCN versus diabetes status in PDAC patients. **(C)** Scatter plot of GDF15 versus SYCN in CP patients. **(D)** Scatter plot of GDF15 versus SYCN in PDAC patients.

In the PDAC group, a positive correlation was observed between serum GDF15 concentrations and bilirubin levels (Spearman’s rho = 0.41; 95% CI: 0.19–0.59; p < 0.001). In the same group, a weak positive point-biserial correlation was observed between SYCN concentration and diabetes status (rpb = 0.28; p = 0.01), corresponding to higher SYCN concentrations in patients with diabetes. In the CP group, serum GDF15 and SYCN levels showed a strong positive correlation (Spearman’s rho = 0.62; 95% CI: 0.48–0.73; p < 0.001), while this correlation was also present, although weaker, in PDAC (Spearman’s rho = 0.36; 95% CI: 0.14–0.56; p = 0.001) and in HCs (Spearman’s rho = 0.47, p < 0.01). The observed correlations are illustrated in the scatter plots presented in **Figure 3**. No significant associations were observed between serum GDF15, SYCN, or TSP-2 levels and other variables, such as PDAC stage, CP Cambridge grade, smoking status, or sex, in the examined groups.

**Figure 4:**
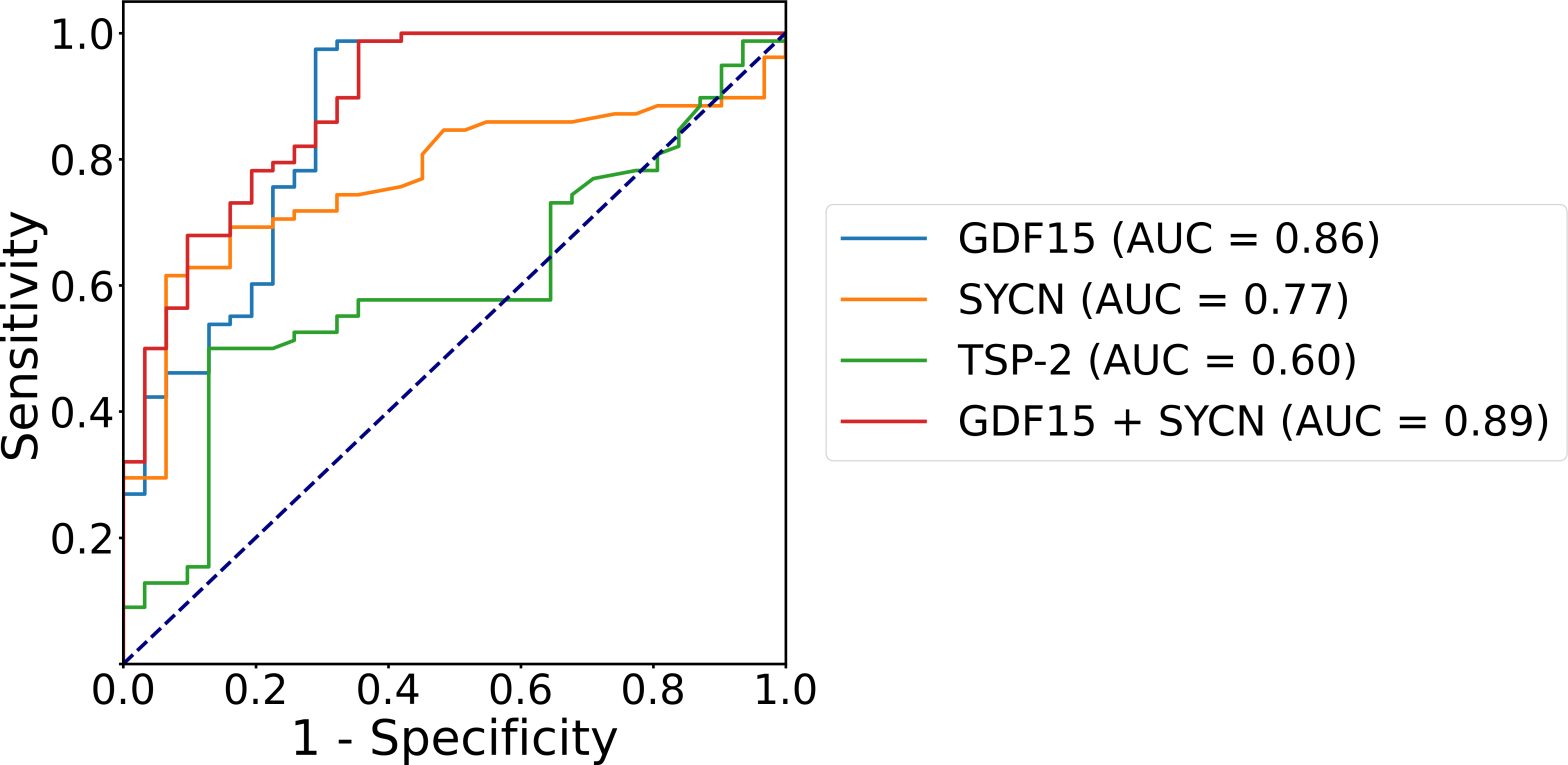
ROC curves for serum GDF15, SYCN, and TSP-2 concentrations in patients with PDAC (n = 78) and healthy controls (HCs; n = 31).

In the differentiation of PDAC from HCs, GDF15 demonstrated the strongest diagnostic performance (AUC = 0.86, 95% CI: 0.80–0.93; Se = 97%, Sp = 71%). In the same comparison, SYCN yielded a lower AUC (0.77, 95% CI: 0.68–0.86) and lower sensitivity (62%) but achieved higher specificity (94%). The best AUC (0.89) was obtained for the combination of GDF15 and SYCN (95% CI: 0.83–0.95; Se = 99%, Sp = 65%). The ROC analysis results are presented in **Figure 4** and **Table 5**.

**Table 5:** Diagnostic performance of individual biomarkers and biomarker panels based on ROC-AUC analysis in differentiating PDAC from HCs.

*ROC analysis parameter*	*GDF15*	*SYCN*	*TSP-2*	*GDF15+SYCN*
*AUC*	0.864	0.769	0.601	0.89
*95% CI*	0.80-0.93	0.68-0.86	0.49-0.71	0.83-0.95
*Optimal ROC threshold*	0.53	0.62	0.69	0.45
*Sensitivity*	97%	62%	50%	99%
*Specificity*	71%	94%	87%	65%
*Mean optimism*	0.0013	−0.0002	0.001	0.004
*Optimism*	0.862	0.769	0.570	0.886
*Bootstrap 95% CI*	0.787-0.954	0.676-0.867	0.486-0.714	0.824-0.960
*Reported cut-off*	5.37	0.14	75.20	0.45

AUC, area under the ROC curve; CI, confidence interval; ROC, receiver operating characteristic; GDF15, growth differentiation factor 15; SYCN, syncollin; TSP-2, thrombospondin-2. Youden index = sensitivity + specificity − 1. Reported cut-off represents biomarker concentration for individual biomarkers and model-derived predicted probability for multivariable panels. Sensitivity and specificity are reported at the Youden-optimal threshold. Bootstrap 95% CI – confidence interval obtained after resampling.

**Figure 5:**
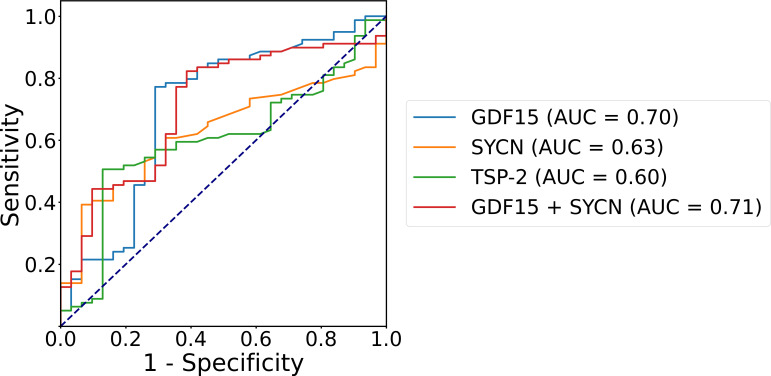
ROC curves for serum GDF15, SYCN, and TSP-2 concentrations in patients with CP (n = 79) versus HCs (n = 31).

ROC curve analysis was further used to comparatively assess the ability of the biomarkers to discriminate between CP and HCs. None of the biomarkers demonstrated strong diagnostic performance in this comparison; the best result was achieved by the combined GDF15 and SYCN panel, with an AUC of 0.71 (95% CI: 0.61–0.81; Se = 82%, Sp = 61%), as shown in **Figure 5** and **Table 6**.

**Table 6:** Diagnostic performance of individual biomarkers and biomarker panels based on ROC-AUC analysis in differentiating CP from HCs.

*ROC analysis parameter*	*GDF15*	*SYCN*	*TSP-2*	*GDF15+SYCN*
*AUC*	0.703	0.628	0.601	0.710
*95% CI*	0.60-0.80	0.52-0.74	0.49-0.71	0.61-0.81
*Optimal ROC threshold*	0.68	0.72	0.71	0.64
*Sensitivity*	77%	39%	51%	82%
*Specificity*	71%	94%	87%	61%
*Mean optimism*	0.002	−0.0005	0.023	0.017
*Optimism*	0.701	0.628	0.578	0.693
*Bootstrap 95% CI*	0.581-0.821	0.528-0.739	0.474-0.687	0.584-0.801
*Reported cut-off*	5.64	0.20	67.60	0.64

AUC, area under the ROC curve; CI, confidence interval; ROC, receiver operating characteristic; GDF15, growth differentiation factor 15; SYCN, syncollin; TSP-2, thrombospondin-2. Youden index = sensitivity + specificity − 1. Reported cut-off represents biomarker concentration for individual biomarkers and model-derived predicted probability for multivariable panels. Sensitivity and specificity are reported at the Youden-optimal threshold. Bootstrap 95% CI – confidence interval obtained after resampling.

**Figure 6:**
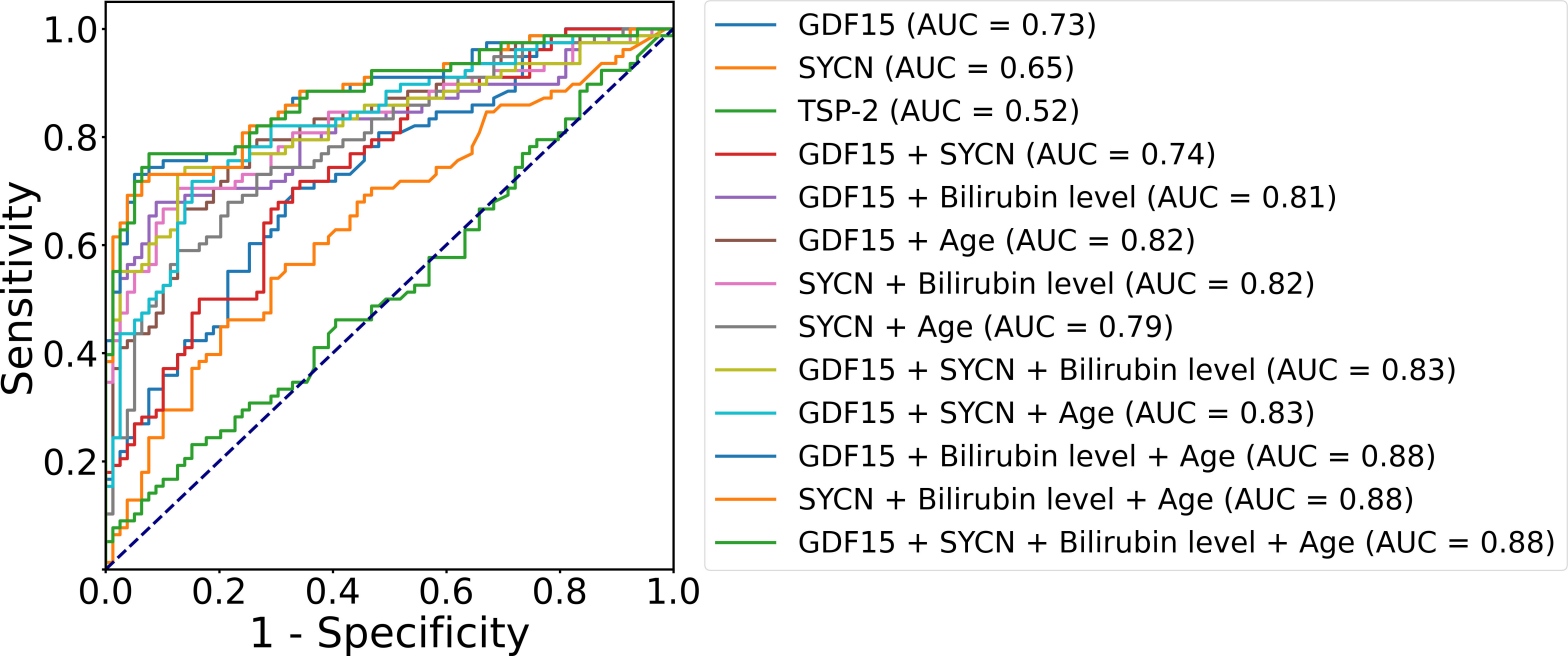
ROC curves for serum GDF15, SYCN, and TSP-2 concentrations in patients with PDAC (n = 78) versus CP (n = 79).

Discriminative performance for differentiating PDAC from CP was also evaluated for each selected biomarker. Individually, GDF15 showed fair diagnostic performance (AUC = 0.73; 95% CI: 0.65–0.81; Se = 71%, Sp = 67%), SYCN achieved weak discriminatory power (AUC = 0.65; 95% CI: 0.56–0.73; Se = 56%, Sp = 68%), and TSP-2 performed at near-chance level (AUC = 0.52; 95% CI: 0.43–0.61; Se = 23%, Sp = 85%). To assess diagnostic performance after adjustment for relevant clinical covariates, multivariable logistic regression models were constructed using selected biomarkers together with bilirubin level and age. The diagnostic performance of these models was then evaluated using ROC curve analysis. After incorporating these variables, diagnostic performance improved, with the highest AUC value of 0.88 obtained for two models: GDF15 + bilirubin level + age (95% CI: 0.82–0.93) and GDF15 + SYCN + bilirubin level + age (95% CI: 0.83–0.94). The model including GDF15, bilirubin, and age showed a sensitivity of 73% and a specificity of 95%, while adding SYCN increased sensitivity to 77% but reduced specificity to 92%. The corresponding ROC curves are shown in **Figure 6**. The remaining multivariable panels, together with biomarker cut-off values for individual biomarkers, are provided in **Supplementary Table 1** in the **Supplementary Materials**.

Bootstrap validation showed only minimal optimism across the analyzed models, supporting the internal stability of the AUC estimates. The strongest discriminatory performance was observed for differentiating PDAC from HCs, particularly for the combined GDF15 + SYCN model, which achieved an optimism-corrected AUC of 0.8860 with a bootstrap 95% CI of 0.8235–0.9598. In the clinically more challenging comparison between CP and PDAC, the addition of clinical variables substantially improved model performance. The model including GDF15, bilirubin level, and age achieved an optimism-corrected AUC of 0.8738 with a bootstrap 95% CI of 0.8202–0.9308, while adding SYCN to this model did not provide further improvement. In contrast, TSP-2 showed weak discriminatory ability across comparisons, with bootstrap confidence intervals frequently including 0.5. The bootstrap validation results are presented in **Tables 5**, **6**; **Supplementary Tables 1**, respectively.

## 4 Discussion

In our study, mean serum GDF15 levels in PDAC were more than fourfold higher than in CP and nearly tenfold higher than in HCs. Similarly, in a large-scale study by Wang et al. (2014) involving 1,472 participants, serum GDF15 concentrations were significantly higher in PDAC than in CP (p < 0.001), or healthy controls (p < 0.001). The same study also demonstrated elevated GDF15 mRNA expression in 51 (81%) of 64 post-surgical cancer tissues using real-time quantitative RT-PCR. However, ROC analyses indicated that serum GDF15 alone was insufficient to reliably distinguish PDAC from either benign pancreatic tumors (AUC = 0.739) or from CP (AUC = 0.592) (22). In our cohort, discriminatory performance of GDF15 between PDAC and CP was slightly better (AUC = 0.73) and improved further when combined with serum bilirubin (AUC = 0.81) or age (AUC = 0.82). We also found that higher serum bilirubin levels (mean 6.48 ± 7.16 mg/dL vs 0.99 ± 0.93 mg/dl, p < 0.001) and older age (mean 69.25 ± 10.39 yrs vs 57.15 ± 12.72 yrs, p < 0,001) were more strongly associated with PDAC than with CP. Data from meta-analysis of 12 studies including over 2,000 patients have shown that serum GDF15 concentrations are elevated in most digestive system tumors and enabled discrimination of healthy individuals and patients with cancer with good accuracy (AUC = 0.84, sensitivity 74% and specificity 83%). Stratification by cancer type revealed strong performance in PDAC, with GDF15 achieving an AUC of 0.80 for discriminating PDAC from healthy subjects and even higher AUC of 0.82 for differentiating between PDAC and CP (39).

In addition, this study revealed a moderate positive correlation between serum GDF15 and bilirubin levels in the PDAC group (r = 0.41, p < 0.001), along with significantly higher serum concentrations of this marker in patients with elevated bilirubin levels (N = 47, Me = 92.15 pg/mL, IQR: 23.57–175.60 vs. N = 31, Me = 15.33 pg/mL, IQR: 10.88–39.01; p < 0.005). This finding is clinically relevant because GDF15 is a pleiotropic stress-response cytokine associated not only with malignancy, but also with inflammatory signaling, tissue stress, metabolic disorders, and broader systemic stress responses (16). Mechanistically, GDF15 expression can be induced in response to cellular stress, inflammatory signaling, tissue damage, hypoxia, and metabolic disturbances, and may be produced by both tumor cells and cells of the tumor microenvironment, including activated macrophages. In PDAC, these processes may coexist with biliary obstruction and cholestasis, making circulating GDF15 a potential marker of combined tumor-related and host stress responses rather than a malignancy-specific signal. Therefore, its elevation in PDAC should not be interpreted as entirely tumor-specific. To our knowledge, no previous study has examined the relationship between GDF15 and bilirubin in PDAC. A prior report demonstrated significantly increased GDF15 levels in patients with cirrhotic PBC compared with non-cirrhotic PBC patients and healthy controls. Importantly, this increase was accompanied by an association between GDF15 and cholestasis-related parameters, including total and direct bilirubin levels (40). Moreover, Liu et al. reported significantly increased serum GDF15 levels in patients with liver cirrhosis and hepatocellular carcinoma compared with healthy controls, further supporting the association of GDF15 with advanced liver disease and malignancy-related systemic stress (20). However, that study did not address the potential influence of jaundice, bilirubin levels, or cholestasis on circulating GDF15 concentrations. Consequently, dedicated studies are needed to determine whether circulating GDF15 levels are primarily influenced by bilirubin/cholestasis-related mechanisms, the malignant process itself, or both. Since none of the patients with CP in the current study presented with jaundice (see **Table 1**), a potential association between GDF15 and cholestasis could not be assessed in this group. The observed correlation between GDF15 and bilirubin in PDAC suggests that cholestasis and systemic stress may partly contribute to increased GDF15 levels, potentially confounding its diagnostic interpretation. This is particularly relevant in the context of PDAC diagnostics, where cholestasis-related hyperbilirubinemia is already recognized as an important confounder of CA19–9 levels and diagnostic performance (41–44). Future studies should therefore include patients with non-malignant biliary obstruction and benign biliary diseases to determine whether GDF15 levels are influenced by cholestasis independently of malignancy.

In the present study, median SYCN levels were higher in the PDAC group than in both CP and HCs, with values more than twofold higher than in CP and more than tenfold higher than in HCs. Makawita et al. showed that SYCN levels in both serum and plasma were significantly higher in patients with PDAC than in healthy controls (both p < 0.005) and in the benign pancreatic disease group, which included intraductal papillary mucinous neoplasms, pancreatic adenomas, tubulovillous adenoma of the duodenum, and pancreatitis samples (p < 0.014). Moreover, in that study, SYCN alone achieved an AUC of 0.74 for discriminating PDAC from healthy controls and also showed utility in differentiating early-stage PDAC (TNM I–II) from healthy subjects, with an AUC of 0.73 or 0.81, depending on the study set (28). In a proteomic-based study by Makawita et al., SYCN was identified as one of the five candidate biomarkers for PDAC diagnosis, and its marked pancreatic tissue specificity was confirmed. The candidates were derived from proteomic profiling of six pancreatic cancer cell lines (BxPc3, MIA-PaCa2, PANC1, CAPAN1, CFPAC1, SU.86.86), and pancreatic juice samples, based on differential expression, overlap, and tissue specificity. Moreover, in the same study, plasma SYCN concentrations were significantly elevated in PDAC patients compared with healthy subjects (p = 0.0011) (29). In our study, the AUC for SYCN alone in distinguishing PDAC from HCs was 0.77. In the exploratory stage-stratified analysis, SYCN concentrations were significantly higher in advanced-stage PDAC compared with HCs and CP after correction for multiple comparisons, whereas the differences between early-stage PDAC and the control groups did not remain significant. No significant difference was observed between early-stage and advanced-stage PDAC. Therefore, these findings should not be interpreted as evidence of a stage-dependent increase in SYCN levels, but rather as an indication that the diagnostic signal of SYCN was more apparent in advanced-stage cases in the present cohort. Further validation in larger cohorts enriched for early-stage PDAC is required to determine the potential utility of SYCN in early disease detection.

In this investigation, serum SYCN concentrations in the PDAC group were significantly higher in patients with diabetes compared with those without diabetes (Me = 1.31 ng/mL vs. 0.23 ng/mL; p < 0.01). In a preclinical model of type 2 diabetes using a leptin-deficient obese mouse strain, isotope label-free quantitative peptidomics revealed increased levels of SYCN, suggesting a potential link with nutritional signals or elevated insulin levels (45). In another study using the INS-1 pancreatic β-cell model, adenoviral-mediated expression of SYCN markedly suppressed regulated insulin secretion stimulated by glucose, glucagon-like peptide-1 (GLP-1), and glibenclamide (46). The reported inhibitory effects of SYCN on insulin exocytosis may indicate its potential involvement in endocrine dysfunction associated with impaired glucose regulation. In the context of PDAC, elevated SYCN levels in patients with diabetes may therefore reflect interactions between metabolic dysregulation, pancreatic endocrine–exocrine crosstalk, and the malignant process. However, it remains unclear whether increased SYCN levels primarily reflect impaired glucose metabolism, endocrine–exocrine pancreatic interactions, the neoplastic process itself, or a combination of these mechanisms. This issue requires further investigation in dedicated mechanistic and clinical studies.

Notably, unlike GDF15, SYCN levels did not correlate with bilirubin and were not significantly elevated in PDAC patients with hyperbilirubinemia compared with those without jaundice (n = 47, Me = 0.45 ng/mL, IQR: 0.05–1.57 vs. n = 31, Me = 0.27 ng/mL, IQR: 0.04–0.82; p = 0.40). To the best of our knowledge, no relationship between SYCN and bilirubin or cholestasis has been reported to date. The available literature on SYCN, particularly regarding its association with malignancies, remains very limited. In a genome-wide DNA methylation study in prostate cancer, Araúzo-Bravo et al. listed SYCN among genes exhibiting hypomethylation at a CpG island region in tumor samples. The authors indicated that this epigenetic feature may have potential utility in distinguishing early malignant lesions from benign changes (47). Considering the known association between PDAC and diabetes, together with the lack of association between SYCN and jaundice in our cohort, SYCN may warrant further evaluation as a biomarker candidate in clinically defined diabetic subgroups. However, its potential relevance for early PDAC detection, particularly in patients with new-onset diabetes, remains hypothetical and could not be verified in the present study. Nevertheless, the observed association between SYCN and diabetes in PDAC patients highlights a potentially potential relevant direction for future studies focused on the metabolic and endocrine–exocrine context of PDAC.

In this study, multivariable panels meaningfully improved discrimination between PDAC and CP compared with single-marker analyses. The two most effective models achieved the same discriminatory performance (AUC = 0.88), but differed in their sensitivity and specificity profiles. The model comprising GDF15, bilirubin, and age achieved a markedly higher specificity (0.95), which may be particularly relevant in the differential diagnosis of PDAC and CP, where reducing false-positive results is of clinically important. The inclusion of SYCN slightly improved sensitivity (0.77 vs. 0.73), but was associated by a modest decrease in specificity (0.92 vs. 0.95). Therefore, while the four-variable model identified a slightly greater proportion of PDAC cases, the GDF15 + bilirubin level + age model may be more suitable when prioritizing specificity in the differential diagnosis of PDAC and CP.

The findings of the current study support the potential utility of GDF15 and SYCN as biomarkers for distinguishing PDAC from both CP and control subjects, which is consistent with our previously published data (48). Of note, a significant positive correlation between GDF15 and SYCN was observed across the examined groups, with the strongest found in CP (r = 0.62), followed by HCs (r = 0.47) and PDAC (r = 0.36). Although no direct molecular interaction between these biomarkers has been reported in the literature, available evidence may support a biologically plausible explanation for this association. GDF15 is a well-established stress-responsive cytokine implicated in PDAC-associated signaling, including activation of the TGF-β/SMAD2/3 pathway (16, 49), whereas SYCN is highly specific to pancreatic acinar cells and has been implicated in regulated exocytosis and zymogen granule membrane organization (26, 50). Therefore, concomitant changes in SYCN and GDF15 may reflect overlapping processes related to pancreatic tissue remodeling, acinar cell dysfunction, inflammatory signaling, and tumor-associated stress responses. However, this interpretation remains hypothetical and requires further mechanistic and clinical validation.

In the present study, TSP-2 showed limited diagnostic value in the context of PDAC. This finding contrasts with previous reports. Kim et al. showed that the TSP-2/CA19–9 panel was superior to CA19–9 alone in discriminating PDAC from CP and intraductal papillary mucinous neoplasms (IPMNs) (51). It is worth noting that, unlike in our study, where serum samples were analyzed, Kim et al. measured both TSP-2 and CA19–9 in plasma. In another study published in 2019, a significant differences in TSP-2 levels was observed between PDAC and CP, as well as between PDAC and high-risk patients with a family history of PDAC (52). In our cohort, however, TSP-2 concentrations did not differ significantly between the groups and showed wide dispersion. This inconsistency may reflect differences in study populations, sample type, assay characteristics, pre-analytical handling, and biological heterogeneity. In addition, in the CP group, serum TSP-2 levels were significantly higher in patients reporting regular alcohol consumption than in those without such history, whereas no analogous association was observed for GDF15 or SYCN. Mechanistically, this association may be related to alcohol-related pancreatic injury, activation of pancreatic stellate cells, and extracellular matrix remodeling. Experimental data from pancreatic injury models indicate that TSP-2 expression increases during pancreatic injury and that pancreatic stellate cells may represent an important source of TSP-2, suggesting its potential role in pancreatic fibrogenesis (53). Therefore, higher TSP-2 levels in CP patients reporting regular alcohol consumption may reflect injury- or fibrosis-related stromal remodeling rather than a PDAC-specific signal. Importantly, regular alcohol consumption was not assessed in the HCs group, which represents an additional limitation and a potential source of unmeasured confounding. Accordingly, the TSP-2 results should be interpreted with caution. Although both cited studies included comparisons with CP, the first did not report CP etiology, whereas the second was conducted in patients with calcifying CP, differing from our cohort, which predominantly included individuals with alcohol-related CP. In addition, neither study reported CP stage, which may partly explain the observed inconsistencies. Further studies including standardized sample processing, detailed assay validation, and stratified analyses according to tumor stage, vascular involvement, stromal characteristics, CP etiology, alcohol exposure, and fibrosis-related features are needed to clarify the diagnostic relevance of TSP-2 in PDAC.

Several limitations of this study should be acknowledged, including the relatively small cohort size, the absence of a formal *a priori* power calculation, and the restriction of the analyses to ELISA-based measurements without complementary genomic investigations. In addition, several biomarkers, clinical variables, and biomarker combinations were evaluated; therefore, the findings should be interpreted with appropriate caution. Although internal validation was performed, external validation in an independent cohort remains necessary before the diagnostic performance of these models can be generalized beyond the studied population. Another limitation is the lack of a direct comparison with CA19-9, which remains the most widely used serum biomarker in the clinical assessment of PDAC. Such a comparison should be included in future studies to determine whether GDF15 and SYCN provide additional diagnostic value beyond CA19-9, either individually or as part of combined biomarker panels. It should also be noted that bilirubin showed strong discriminatory performance and may partly reflect biliary obstruction rather than a malignancy-specific biomarker signal. Therefore, diagnostic models including bilirubin should be interpreted cautiously, particularly in cohorts with a high proportion of patients with advanced and/or obstructive disease. Furthermore, differences in alcohol consumption history among patients with CP may have influenced circulating TSP-2 levels and potentially attenuated its apparent diagnostic value for distinguishing PDAC from chronic pancreatitis. This potential confounding factor was not fully controlled for in the present study and should be addressed in future validation cohorts. Nevertheless, the primary aim of this study was to identify clinically feasible biomarkers that can be quantified using widely available assays, thereby facilitating potential implementation in routine laboratory practice.

PDAC remains exceptionally difficult to diagnose because of the lack of specific, clinically validated biomarkers and the limitations of CA19-9 (54). In our study, SYCN appears particularly interesting, as its concentrations were significantly higher in the overall PDAC group than in HCs and CP, while in the exploratory stage-stratified analysis the significant differences were observed mainly for advanced-stage PDAC compared with HCs and CP. Importantly, SYCN levels did not differ significantly between early-stage and advanced-stage PDAC and were not associated with hyperbilirubinemia. SYCN also showed high specificity of 94% in distinguishing PDAC from HCs. The absence of a significant difference in SYCN levels between HCs and patients with CP suggests that, in this cohort, SYCN elevation was not primarily driven by CP; however, whether this reflects PDAC-specific biology requires further investigation. These findings suggest that SYCN deserves further investigation as a component of biomarker panels, particularly in clinically relevant settings such as patients with new-onset diabetes, including approaches based on the END-PAC model and screening strategies assessed in the PANDOME Study (55, 56). The relationship between diabetes and PDAC is complex and remains incompletely understood (57). The incorporation of SYCN could potentially improve the selection of individuals requiring further diagnostic evaluation, such as EUS, although this remains hypothetical and should be validated in cohorts specifically designed to assess early-stage PDAC and high-risk populations.

The differentiation between PDAC and non-malignant pancreatic diseases such as CP remains a major clinical challenge. Imaging findings may be inconclusive, and biopsy-based histopathology can be affected by sampling error and tumor heterogeneity, thereby contributing to diagnostic uncertainty and delayed diagnostic clarification. Our results provide an initial assessment of the diagnostic potential of GDF15 and SYCN, particularly when combined with relevant clinical variables, and suggest that these biomarkers may support diagnostic assessment and risk stratification. Nevertheless, GDF15 and SYCN should be considered potential complementary biomarkers rather than direct replacements for established diagnostic markers. Their potential role in earlier-stage PDAC and in differentiation from CP requires further evaluation in appropriately designed cohorts, including direct comparison with established biomarkers such as CA19-9. Since PDAC and CP may present with overlapping clinical and radiological features, simple biomarkers combined with routinely available clinical parameters may represent a promising direction for future diagnostic models in carefully characterized patient populations.

## Data Availability

The raw data supporting the conclusions of this article will be made available by the authors, without undue reservation.
