# Supplementary material for: Macrophage inhibitory cytokine 1, syncollin and thrombospondin-2 in pancreatic ductal adenocarcinoma and chronic pancreatitis differentiation
Source: Front Oncol. 2026 Jun 26;16:1866001. doi: 10.3389/fonc.2026.1866001 (PMC13349920; doi:10.3389/fonc.2026.1866001)
Supplement: Supplementary file 1 [file Table1.docx]

**Table 7.** Diagnostic performance of individual biomarkers and biomarker panels based on ROC-AUC analysis in differentiating PDAC from CP.
***Note:*** *AUC – area under the ROC curve; CI – confidence interval; ROC – receiver operating characteristic; GDF15 – growth differentiation factor 15; SYCN – syncollin; TSP-2 – thrombospondin-2. Youden index = sensitivity + specificity − 1. Reported cut-off represents biomarker concentration for individual biomarkers and model-derived predicted probability for multivariable panels. Sensitivity and specificity are reported at the Youden-optimal threshold. Bootstrap 95% CI – confidence interval obtained after resampling.*

| ROC analysis parameter | GDF15 | SYCN | TSP-2 | GDF15 +SYCN | GDF15 +BiL | GDF15 +Age | SYCN + BiL | SYCN + Age | GDF15 +SYCN +BiL | GDF15 +SYCN +Age | GDF15 +BiL +Age | SYCN +BiL +Age | GDF15 +SYCN +BiL  +Age |
| --- | --- | --- | --- | --- | --- | --- | --- | --- | --- | --- | --- | --- | --- |
| **AUC** | 0.723 | 0.646 | 0.520 | 0.739 | 0.815 | 0.821 | 0.823 | 0.791 | 0.829 | 0.826 | 0.880 | 0.878 | 0.881 |
| **95% CI** | 0.65-0.81 | 0.56-0.73 | 0.43-0.61 | 0.66-0.82 | 0.75-0.88 | 0.75-0.89 | 0.76-0.89 | 0.72-0.86 | 0.76-0.89 | 0.76-0.89 | 0.82-0.93 | 0.82-0.93 | 0.83-0.94 |
| **Optimal ROC threshold** | 0.40 | 0.46 | 0.48 | 0.38 | 0.44 | 0.47 | 0.39 | 0.52 | 0.40 | 0.54 | 0.56 | 0.54 | 0.51 |
| **Sensitivity** | 71% | 56% | 23% | 72% | 68% | 79% | 71% | 73% | 74% | 72% | 73% | 73% | 77% |
| **Specificity** | 67% | 68% | 85% | 66% | 91% | 73% | 87% | 73% | 86% | 85% | 95% | 92% | 92% |
| **Mean optimism** | 0.002 | −0.0005 | 0.023 | 0.017 | 0.0003 | 0.002 | 0.006 | 0.005 | 0.008 | 0.005 | 0.006 | 0.005 | 0.090 |
| **Optimism** | 0.701 | 0.628 | 0.578 | 0.693 | 0.7296 | 0.818 | 0.817 | 0.790 | 0.821 | 0.821 | 0.874 | 0.873 | 0.872 |
| **Bootstrap 95% CI** | 0.581-0.821 | 0.528-0.739 | 0.474-0.687 | 0.584-0.801 | 0.659-0.811 | 0.750-0.883 | 0.753-0.886 | 0.720-0.854 | 0.757-0.892 | 0.763-0.885 | 0.820-0.931 | 0.819-0.929 | 0.822-0.930 |
| **Youden cut-off** | 5.64 | 0.20 | 67.60 | 0.38 | 0.44 | 0.47 | 0.39 | 0.52 | 0.4 | 0.54 | 0.56 | 0.54 | 0.51 |
